# Supplementary material for: Metabolic Basis of Post-Infectious Sequelae After Ebola Virus Disease
Source: medRxiv. 2026 Jan 6:2026.01.02.25343095. Preprint. [Version 1] doi: 10.64898/2026.01.02.25343095 (PMC12803395; doi:10.64898/2026.01.02.25343095)
Supplement: Supplement 1 [file media-1.pdf]

Supplementary Data

Supplementary Tables

Sample Collection Date (MM-YYYY)	Clinical Sequelae Date (MM-YYYY)	Sequelae
03-2016	03-2016	MSK/GI
02-2017	03-2016	MSK/GI
03-2016	03-2016	MSK/GI
03-2016	03-2016	MSK/GI
03-2016	03-2016	CP
02-2017	03-2016	MSK/GI
08-2018	03-2022	CP
04-2019	04-2019	CP
03-2016	03-2016	MSK/GI
02-2017	02-2017	MSK/GI
04-2019	04-2019	CP
04-2019	03-2020	CP
08-2018	03-2022	CP
03-2016	03-2016	MSK/GI
03-2017	03-2017	MSK/GI
03-2017	03-2017	CP
04-2019	04-2019	CP
03-2017	03-2017	MSK/GI
09-2016	09-2016	MSK/GI
03-2016	03-2016	MSK/GI
04-2019	03-2020	CP
04-2019	03-2020	CP

10-2016	04-2017	CP
04-2017	04-2017	CP
04-2017	04-2017	CP
04-2017	04-2017	CP
04-2016	04-2016	CP
04-2016	04-2017	CP
04-2016	04-2016	MSK/GI
03-2016	08-2016	CP
08-2016	08-2016	CP
07-2017	07-2017	MSK/GI
11-2017	11-2017	MSK/GI
11-2017	11-2017	MSK/GI
12-2017	11-2017	MSK/GI
12-2017	11-2017	MSK/GI
09-2018	03-2020	CP

Supplementary Table 1: Dates of plasma collection and sequelae for all survivors with sequelae. ID not shown to protect patient privacy.

	**PES (n=37)**	**Asymptomatic Survivors (n=20)**	**p-value**	**PES & Asymptomatic Survivor (n=57)**
**Median age (y)**	30	28	0.22	29
**Female (%)**	59.5	60	0.99	59.6
**Median BMI (kg/m^2)**	24.5	22.1	0.11	23.1
**Median Sample Collection Date**	2017-03-08	2017-11-26	0.11	2017-11-21

	**PES (n=37)**	**Household Controls (n=20)**	**p-value**	**PES & Household Controls (n=57)**
**Median age (y)**	30	29.5	0.75	30
**Female (%)**	59.5	60.0	0.98	59.6
**Median BMI (kg/m^2)**	24.5	23.5	0.49	24.4
**Median Sample Collection Date**	2017-03-08	2017-07-14	0.50	2017-04-24
	**MSK/GI (n=18)**	**CP (n=19)**	**p-value**	**MSK/GI & CP (n=37)**
**Median age (y)**	30	25	0.82	30
**Female (%)**	66.7	52.6	0.89	59.5
**Median BMI (kg/m^2)**	27.2	22.7	0.11	24.5
**Median Sample Collection Date**	2017-02-12	2017-04-24	0.02*	2017-03-08

Supplementary Table 2: Logistic regression included age, sex, BMI and sample collection date to predict group membership. No characteristic was significant in predicting PES when comparing EVD survivors with PES and asymptomatic survivors or EVD survivors with PES and household controls. EVD survivors with cardiopulmonary (CP) sequelae had a significantly later collection date compared to survivors with musculoskeletal or gastrointestinal (MSK/GI) sequelae. Logistic regression performed in R Studio. PES= Post-Ebola syndrome, EVD= Ebola virus disease.

	FC	log2(FC)	p.adjusted	-log10(p)
Acrylate	4.0574	2.0206	1.83E-05	4.7381
Acetate	3.6465	1.8665	1.83E-05	4.7381
3-Hydroxy-3-methylglutarate	3.6228	1.8571	1.83E-05	4.7381
Glucose	2.9963	1.5832	1.83E-05	4.7381
Malate	0.45773	-1.1274	9.78E-05	4.0099
2-Oxo-4-hydroxy-4-carboxy-5-ureidoimidazoline	4.6347	2.2125	0.00010336	3.9857
Succinate	0.45074	-1.1496	0.00010336	3.9857
Methylmalonate	0.45074	-1.1496	0.00010336	3.9857
Threonate-1,4-lactone	0.45074	-1.1496	0.00010336	3.9857
Propanoate	0.48027	-1.0581	0.00043485	3.3617
Oxalosuccinate	7.7896	2.9615	0.0016024	2.7952
Palmitoylcarnitine	0.38619	-1.3726	0.0022608	2.6457
Ascorbate	0.16542	-2.5958	0.0036576	2.4368
N-Acetylornithine	0.017035	-5.8753	0.0068859	2.162
Deoxycytidine	0.14416	-2.7942	0.0068859	2.162
Hypoxanthine	0.26767	-1.9015	0.0076167	2.1182
2-Hydroxyhippurate	0.38912	-1.3617	0.0080669	2.0933
Dopaquinone	0.38912	-1.3617	0.0080669	2.0933
Leucodopachrome	0.38912	-1.3617	0.0080669	2.0933
alpha-Hydroxyhippurate	0.38912	-1.3617	0.0080669	2.0933
4- Carboxyphenylglycine	0.38912	-1.3617	0.0080669	2.0933
Dihydrouracil	0.39851	-1.3273	0.0080669	2.0933
gamma-Glutamyl-2-aminobutyrate	0.035676	-4.8089	0.0084969	2.0707
Cysteinylglycine	0.23083	-2.1151	0.0084969	2.0707
2-Oxoglutarate	0.21137	-2.2421	0.010247	1.9894
Inosine-5'-monophosphate (IMP)	0.4141	-1.2719	0.014766	1.8307
6-Phospho-gluconate	0.21403	-2.2241	0.015814	1.801
Carnosine	0.25697	-1.9603	0.024622	1.6087
Diethanolamine	2.7817	1.476	0.030025	1.5225
Methyluridine	0.38369	-1.382	0.030025	1.5225
Aspartate	2.6075	1.3827	0.035226	1.4531
4-Fumarylacetoacetate	0.49123	-1.0255	0.039728	1.4009
4-Maleylacetoacetate	0.49123	-1.0255	0.039728	1.4009
Uridine monophosphate (UMP)	0.45329	-1.1415	0.042379	1.3728

Supplementary table 3: Fold change (FC) and adjusted p-values (false discovery rate (FDR) from Benjamini-Hochberg (BH) method) for 34 significantly differential metabolites between EVD survivors with PES and asymptomatic survivors.

Variable	by Variable	Spearman ρ	Prob>|ρ|	BH.corr.pvalue
Succinate	Glucose	-0.5960	0.0001	0.00065941
Methylmalonate	Glucose	-0.5960	0.0001	0.00065941
Methylmalonate	Succinate	1	0.0001	0.00065941
Succinate	Malate	0.4407	0.0063	0.02370508
Methylmalonate	Malate	0.4407	0.0063	0.02370508
Glucose	Malate	-0.4066	0.0125	0.04141791
Threonate-1,4-lactone	Glucose	-0.5960	0.0001	0.00065941
Threonate-1,4-lactone	Succinate	1	0.0001	0.00065941
Threonate-1,4-lactone	Methylmalonate	1	0.0001	0.00065941
Acrylate	Glucose	0.9381	0.0001	0.00065941
Deoxycytidine	Malate	0.5861	0.0001	0.00065941
N-Acetylornithine	Glucose	-0.6449	0.0001	0.00065941
Inosine-5'-monophosphate (IMP)	Glucose	-0.6361	0.0001	0.00065941
Propanoate	Succinate	0.826	0.0001	0.00065941
Propanoate	Methylmalonate	0.826	0.0001	0.00065941
2-Oxo-4-hydroxy-4-carboxy-5-ureidoimidazoline	Glucose	0.7892	0.0001	0.00065941
3-Hydroxy-3-methylglutarate	Glucose	0.8888	0.0001	0.00065941
Acetate	Glucose	0.9263	0.0001	0.00065941
Acetate	Succinate	-0.5846	0.0001	0.00065941
Acetate	Methylmalonate	-0.5846	0.0001	0.00065941
6-Phospho-gluconate	Succinate	0.6264	0.0001	0.00065941
6-Phospho-gluconate	Methylmalonate	0.6264	0.0001	0.00065941
Acrylate	Succinate	-0.5799	0.0002	0.00122202
Acrylate	Methylmalonate	-0.5799	0.0002	0.00122202
2-Oxo-4-hydroxy-4-carboxy-5-ureidoimidazoline	Succinate	-0.5778	0.0002	0.00122202
2-Oxo-4-hydroxy-4-carboxy-5-ureidoimidazoline	Methylmalonate	-0.5778	0.0002	0.00122202
N-Acetylornithine	Succinate	0.5614	0.0003	0.00175263
N-Acetylornithine	Methylmalonate	0.5614	0.0003	0.00175263
3-Hydroxy-3-methylglutarate	Succinate	-0.5372	0.0006	0.00327541
3-Hydroxy-3-methylglutarate	Methylmalonate	-0.5372	0.0006	0.00327541
2-Hydroxyhippurate	Succinate	0.5332	0.0007	0.00335396
2-Hydroxyhippurate	Methylmalonate	0.5332	0.0007	0.00335396
Dopaquinone	Succinate	0.5332	0.0007	0.00335396

Dopaquinone	Methylmalonate	0.5332	0.0007	0.00335396
Leucodopachrome	Succinate	0.5332	0.0007	0.00335396
Leucodopachrome	Methylmalonate	0.5332	0.0007	0.00335396
alpha-Hydroxyhippurate	Succinate	0.5332	0.0007	0.00335396
alpha-Hydroxyhippurate	Methylmalonate	0.5332	0.0007	0.00335396
4- Carboxyphenylglycine	Succinate	0.5332	0.0007	0.00335396
4- Carboxyphenylglycine	Methylmalonate	0.5332	0.0007	0.00335396
6-Phospho-gluconate	Glucose	-0.4844	0.0024	0.01031226
2-Oxo-4-hydroxy-4-carboxy-5-ureidoimidazoline	Malate	-0.4704	0.0033	0.01382264
Deoxycytidine	Glucose	-0.4535	0.0048	0.01891598
Uridine monophosphate (UMP)	Glucose	-0.4538	0.0048	0.01891598
Threonate-1,4-lactone	Malate	0.4407	0.0063	0.02370508
6-Phospho-gluconate	2-Oxoglutarate	-0.4391	0.0066	0.02455642
Cysteinylglycine	Malate	0.4365	0.0069	0.02538895
3-Hydroxy-3-methylglutarate	Malate	-0.4367	0.0069	0.02538895
Hypoxanthine	Glucose	-0.4334	0.0074	0.02693115
3-Hydroxy-3-methylglutarate	Oxalosuccinate	0.4294	0.008	0.02895652
Acrylate	Malate	-0.4286	0.0081	0.02900323
2-Oxo-4-hydroxy-4-carboxy-5-ureidoimidazoline	Oxalosuccinate	0.4229	0.0091	0.03156563
Acetate	Malate	-0.4229	0.0091	0.03156563
Acrylate	Oxalosuccinate	0.408	0.0122	0.040626
Acetate	Oxalosuccinate	0.3988	0.0145	0.04710732
Glucose	Diethanolamine	0.674	0.0001	0.00065941
Succinate	gamma-Glutamyl-2-aminobutyrate	0.5863	0.0001	0.00065941
Methylmalonate	gamma-Glutamyl-2-aminobutyrate	0.5863	0.0001	0.00065941
2-Oxoglutarate	Ascorbate	0.811	0.0001	0.00065941
2-Oxoglutarate	4-Fumarylacetoacetate	0.7044	0.0001	0.00065941
2-Oxoglutarate	4-Maleylacetoacetate	0.7044	0.0001	0.00065941
Malate	Ascorbate	0.5538	0.0004	0.00225763
Glucose	gamma-Glutamyl-2-aminobutyrate	-0.5555	0.0004	0.00225763
2-Oxoglutarate	Carnosine	-0.5344	0.0007	0.00335396
Succinate	Carnosine	0.4919	0.002	0.00888
Methylmalonate	Carnosine	0.4919	0.002	0.00888
Succinate	Diethanolamine	-0.4689	0.0034	0.01389202

Methylmalonate	Diethanolamine	-0.4689	0.0034	0.01389202
Glucose	Carnosine	-0.4244	0.0089	0.0313619

Supplementary Table 4: Spearman ρ values for metabolites related to TCA cycle or glucose. Metabolites considered related to the TCA cycle and that had significant interactions included succinate, malate, methylmalonate, oxalosuccinate, 2-oxoglutarate. Only correlations with a BH corrected p value of <.05 were included.

**Component 1**		
**Metabolite**	**VIP Score**	**Significance - PES**
Acetate	2.6838	Upregulated
Acrylate	2.6663	Upregulated
Glucose	2.6593	Upregulated
3-Hydroxy-3-methylglutarate	2.6552	Upregulated
Malate	2.5089	Downregulated
2-Oxo-4-hydroxy-4-carboxy-5-ureidoimidazoline	2.4658	Upregulated
Succinate	2.4551	Downregulated
Methylmalonate	2.4551	Downregulated
Threonate-1,4-lactone	2.4551	Downregulated
Propanoate	2.3163	Downregulated
LysoPA(16:0/0:0)	2.2934	Not significant
Lyxose	2.1664	Not significant
Oxalosuccinate	2.1586	Upregulated

Tyramine	2.1061	Not significant
Phenylethanolamine	2.1061	Not significant
**Component 2**		
**Metabolite**	**VIP Score**	**Significance - PES**
4-Hydroxy-enol-phenylpyruvate	2.3796	Not significant
4-Hydroxyphenylpyruvate	2.3796	Not significant
Caffeate	2.3796	Not significant
Acetate	2.3736	Upregulated
Acrylate	2.3626	Upregulated
Glucose	2.3488	Upregulated
3-Hydroxy-3-methylglutarate	2.3391	Upregulated
Malate	2.2013	Downregulated
Succinate	2.1881	Downregulated
Methylmalonate	2.1881	Downregulated
Threonate-1,4-lactone	2.1881	Downregulated
2-Oxo-4-hydroxy-4-carboxy-5-ureidoimidazoline	2.1641	Upregulated
Palmitoylcarnitine	2.125	Downregulated
LysoPA(16:0/0:0)	2.1045	Not significant
Propanoate	2.0713	Downregulated

Supplementary Table 5: Top 15 variables of importance (VIP) for PLS-DA model. Metabolite considered significantly upregulated or downregulated if FC > 2.0 and FDR <.05 when comparing PES and asymptomatic survivor groups.

Supplementary Figures

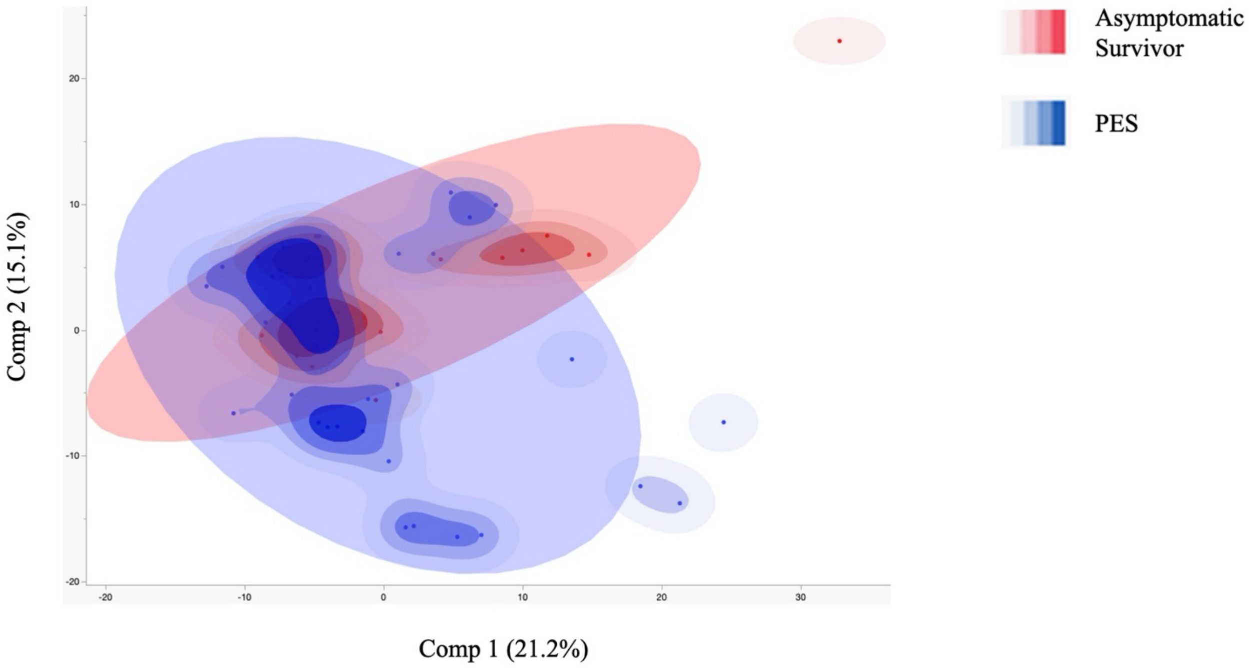

Supplementary Figure 1: Principal Components Analysis (PCA) Demonstrates Separation of Survivors with PES and Asymptomatic Survivors

EVD survivors with PES separate from asymptomatic survivors based on metabolic profile. Data analyzed in MetaboAnalyst and visualized in JMP.

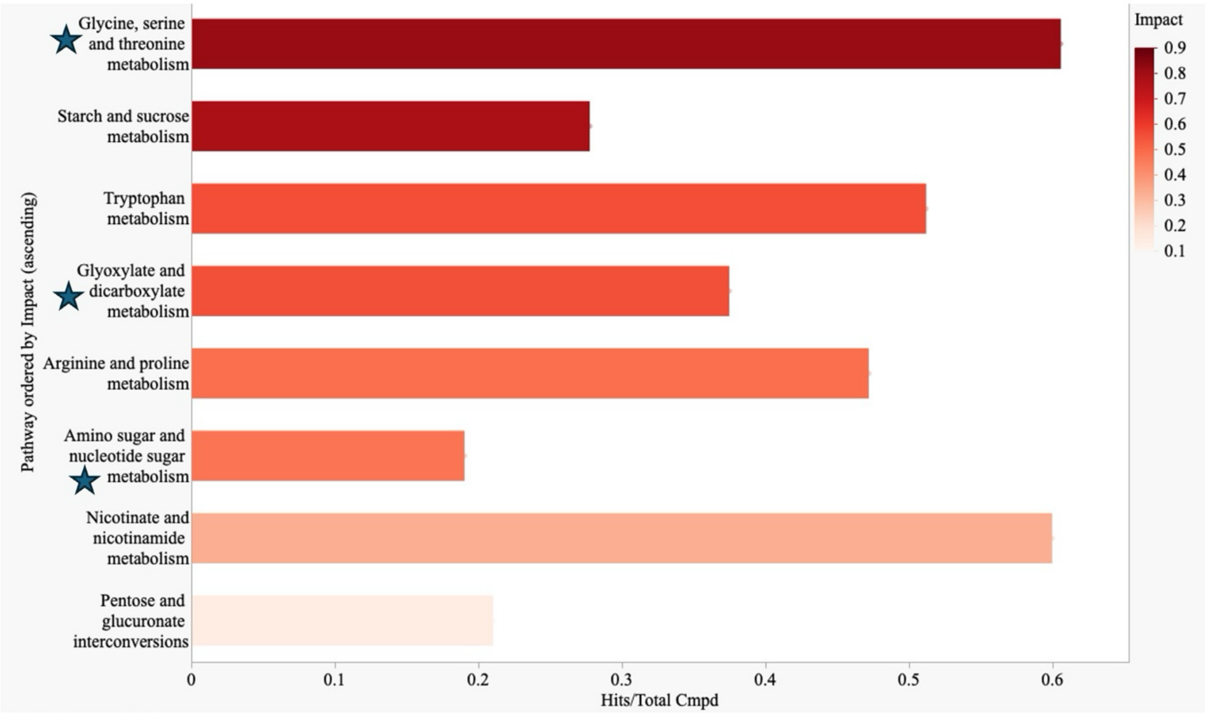

Supplementary Figure 2: Several Metabolic Pathways are Significantly Upregulated or Tied in EVD Survivors with PES Compared to Asymptomatic Survivors

Pathways with an equal number of upregulated and downregulated ("tie" pathways) metabolites are denoted by a star. Pathways are ordered by impact score. Impact score required to be >0.2 and FDR <.05 for inclusion. Pathway analysis performed in MetaboAnalyst and data visualized using JMP.

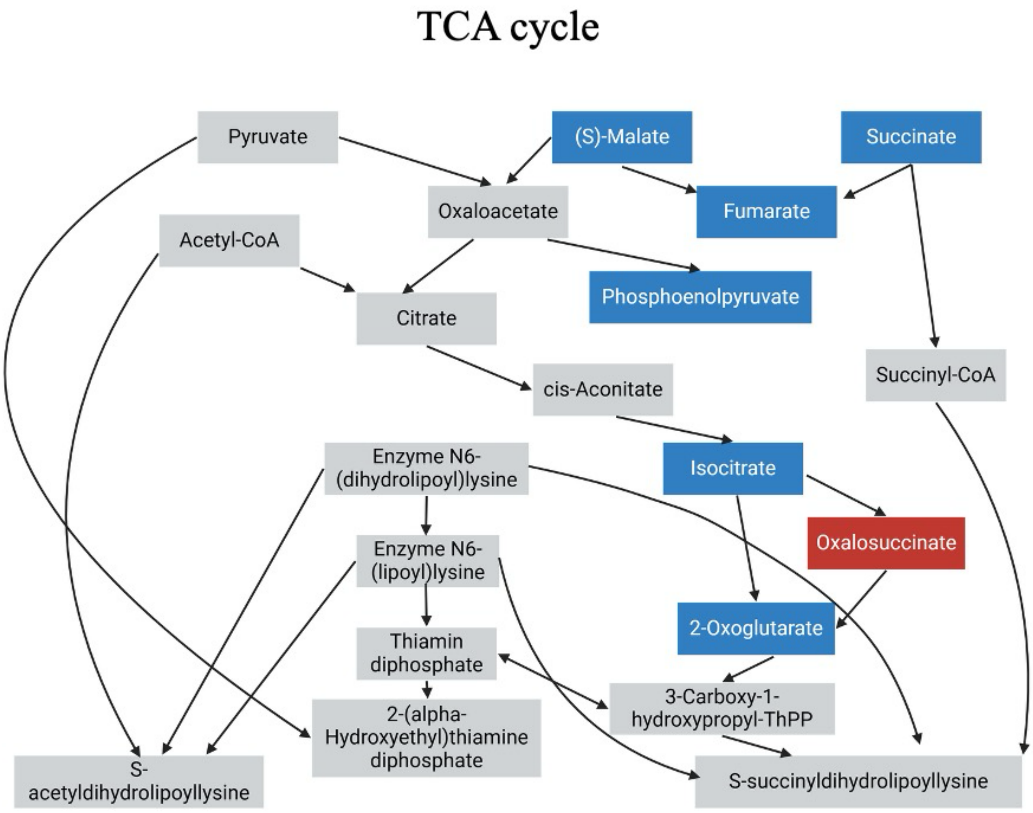

Supplementary Figure 3: EVD Survivors with PES Demonstrates Primarily Downregulated Metabolism of the TCA Cycle Compared to Asymptomatic Survivors

Significantly downregulated metabolites are highlighted in blue. Significantly upregulated metabolites are highlighted in red. Metabolites that were neither significantly upregulated or downregulated are in grey. Pathway analysis performed in MetaboAnalyst. Created in BioRender. Sanford, A. (2026) https://BioRender.com/v1jj72h

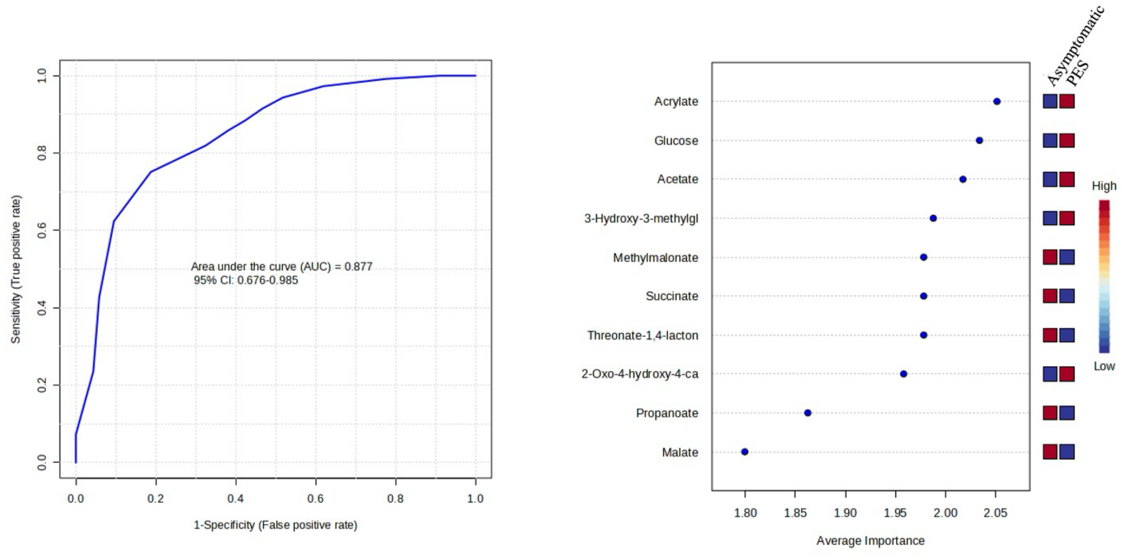

A

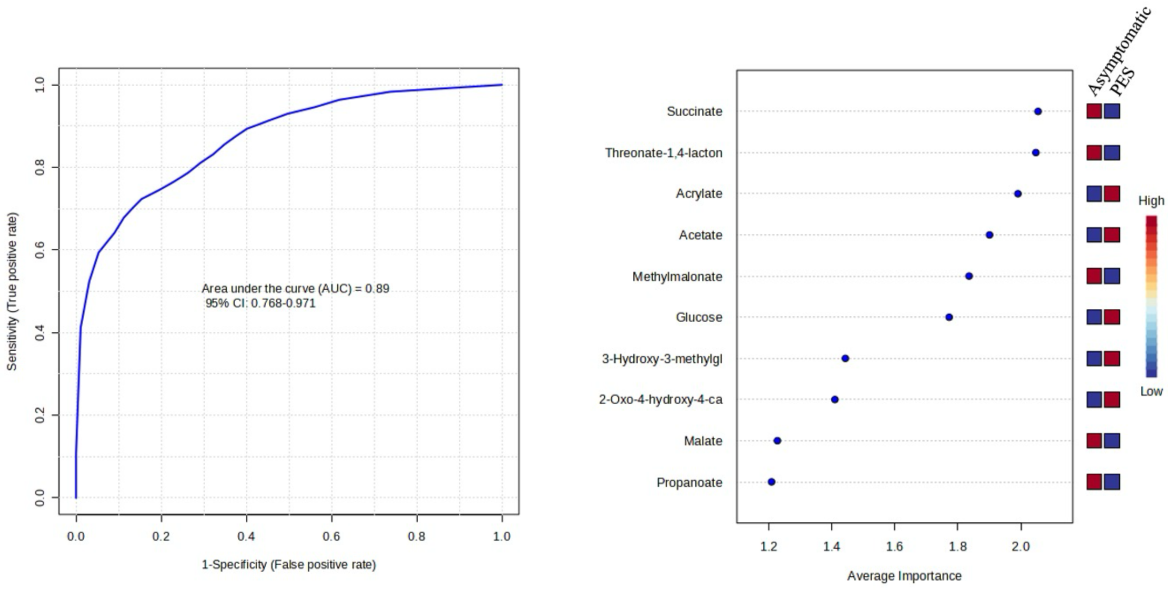

B

Supplementary Figure 4: Discovery Models of Biomarkers of PES

Biomarker analysis was performed to identify group membership (PES vs asymptomatic survivor) in MetaboAnalyst. Discovery (n= 39) and validation (n= 18) cohorts were used (discovery cohorts shown here, validation cohorts in Figures 5A-B). Potential biomarkers were selected from PLS-DA and Random Forest (RF) modeling in the discovery cohort. The top 10 most important features for predicting PES were the same in RF and PLS-DA modeling. If the metabolite was higher in the PES or asymptomatic group, the box to the right of the figure was marked red, and if that metabolite was lower, the box was marked blue. Figure 4A:

ROC from discovery model from PLS-DA and 10 most important features, ranked by importance. Figure 4B: ROC from discovery model from RF and 10 most important features, ranked by importance.
